# Medical Statistics

**Published:** 1837-04

**Authors:** 


### MEDICAL STATISTICS.

### *On the Prevalence of Consumption in the United States of America.* By AMARIAH BRIGHAM, M.D.

THE great mortality from this disease in the United States, and the general inutility of remedial efforts, ought to excite public attention, and awaken enquiry as to methods of prevention. From an examination of the bills of mortality within my reach, and information derived from medical correspondents in various sections of the Union, I am obliged to conclude that there are at least fifty thousand deaths by consumption, every year, in this country.

Allowing the same mortality from this disease to the entire population of the United States that there has been from it for several years past in the cities of Portsmouth, Boston, New York, Philadelphia, Baltimore, Washington, Charleston, and Natchez, and the number of deaths for the whole country would considerably exceed fifty thousand.

In this communication, I purpose to exhibit, by the statement of a few facts, the alarming prevalence of this disease in the United States, with the hope of awakening attention to some measures calculated to diminish it.

The following table exhibits for a series of years the number of deaths by consumption, and also the whole number of deaths from all diseases, in some of the largest cities in the country:

| | New York. | | Philadelphia. | | Boston. | | Baltimore. | | Washington. | | Charleston. | | Portsmouth. | |
|---|---|---|---|---|---|---|---|---|---|---|---|---|---|---|
| Years. | Whole No. of Deaths. | By Consumption. | Whole No. of Deaths. | By Consumption. | Whole No. of Deaths. | By Consumption. | Whole No. of Deaths. | By Consumption. | Whole No. of Deaths. | By Consumption. | Whole No. of Deaths. | By Consumption. | Whole No. of Deaths. | By Consumption. |
| 1824 | 4341 | 736 | 4399 | 576 | 1297 | 242 | 1468 | 188 | .... | .... | 1059 | 101 | 118 | 22 |
| 1825 | 5018 | 843 | 3812 | 519 | 1450 | 220 | 1545 | 295 | .... | .... | 840 | 152 | 109 | 28 |
| 1826 | 4973 | 820 | 4151 | 587 | 1254 | 231 | 1922 | 306 | 283 | 27 | 764 | 124 | 171 | 27 |
| 1827 | 5181 | 829 | 3945 | 523 | 1022 | 178 | 1498 | 267 | 251 | 27 | .... | .... | 132 | 23 |
| 1828 | 5181 | 906 | 4292 | 581 | 1233 | 217 | 1702 | 295 | 254 | 37 | .... | .... | 121 | 24 |
| 1829 | 5094 | 880 | 4293 | 638 | 1221 | 203 | 1849 | 267 | 304 | 31 | 762 | 412 | 121 | 28 |
| 1830 | 5537 | 974 | 4250 | 636 | 1125 | 193 | 2086 | 332 | 339 | 46 | .... | .... | 178 | 24 |
| | 35,325 | 5,988 | 29,142 | 4,060 | 8,602 | 1,484 | 12,070 | 19,50 | 1,431 | 168 | 3,425 | 489 | 950 | 176 |

From this table it appears, that the proportion of deaths by consumption to the whole of the deaths by all diseases is, at

| | | | |
|---|---|---|---|
| Portsmouth, N. H., | as 1 in 5,39 | Philadelphia, | as 1 in 7,17 |
| Boston, | " 1 " 5,79 | Baltimore, | " 1 " 6,18 |
| New York, | " 1 " 5,89 | Washington, | " 1 " 8,51 |

Charleston, S. C., as 1 in 7,08

It also appears that the mortality from consumption is the greatest in the most northern cities. Thus it is greater at Portsmouth than at Boston, and it is greater at Portland than at Portsmouth. The whole number of deaths from all diseases during the last year at Portland was 305, of which number, eighty-four were by consumption, being in proportion to all the deaths as one in 3,53. The disease is more frequent in Boston than in New York, and more so in New York than in the cities farther south. It will be noticed, however, that the deaths by this disease are less in Philadelphia than in Charleston and Baltimore. This may be owing partly to the inland situation of Philadelphia; as it is well established, that this disease is more prevalent in the cities on the Atlantic, than in those of the interior; and partly to the fact, that many individuals from the north, affected with disease of the lungs, visit the cities of the south, particularly Charleston, for the sake of a warm climate, but die there of consumption. This prevents our ascertaining from bills of mortality all the deaths that occur from consumption among the inhabitants of the northern cities, and improperly swells the list of reported deaths in some of the southern cities.

But though there is less of this disease in southern and warm climates than in northern and cold ones, yet it prevails to a great extent even in warm countries—in Italy, in the West Indies, and in the southern states of this country. As I have said, it prevails more in towns on the Atlantic than in those of the interior, though the difference is not, I apprehend, so great as many suppose. According to the statements of some writers, there is but little of this disease in the Valley of the Mississippi, and in the western parts of this country. Mr. Flint, in his "History and Geography of the Mississippi Valley," remarks that "Pulmonary consumption is a very uncommon disease, not often witnessed in the northern region of the western country. Fifty persons fall victims to this terrible destroyer in the Atlantic country to one that dies of it here." We can hardly believe this to be correct. It is difficult, however, to obtain much accurate information on this subject, as but few of the towns in the western country have published bills of mortality; but from information derived from various sources deemed authentic, it appears that although this disease is less frequent at the west than in the east, yet it prevails even in the former to a great extent.

The whole number of deaths at Natchez for thirteen years—from 1822 to 1835—was 1904, and the deaths by consumption during the same time 100. This at first appears to be but a slight mortality from consumption. But it should be recollected that the total mortality is very great—equal to that of the most sickly cities of Europe; and that 100 deaths by consumption in thirteen years, for the population of Natchez, is nearly equal to the mortality from this disease in Philadelphia—though it is in proportion to the whole number of deaths but as one in nineteen, while in Philadelphia the proportion is as one in seven.

It is however true, that many predisposed to consumption, and while in the Atlantic country, are affected with lung complaints, regain their health on removing to the west. Several such instances have fallen within my own observation. But this may as properly be attributed to the remedial effect of a long journey and mental excitement, as to the climate of the western country. Long journeys, with pleasurable mental excitement, are among the most useful remedies in the early stages of this disease.

It is generally believed that consumption is much more frequent and fatal in cities than in the country. This is probably true in Europe, where the inhabitants of the large cities are far less healthy than those in the country. But from all the facts I can obtain on this subject in the United States, it appears there is not great, though there is some difference. It is difficult to ascertain the exact amount of mortality in most of the small towns of this country, as but few of them have any bills of mortality. In the town of Woodbury, in the western part of Connecticut, containing 2050 inhabitants, the whole number of deaths during the last eleven years is 347, of which number fifty-five, or about one-sixth of the whole, were by consumption. I have accurate accounts from above twenty small towns in the interior of Connecticut and the western part of Massachusetts, some but for one and others for three and four years, which show that from one-sixth to one-eighth of all the deaths are by this disease. By the bill of mortality for Rutland (East Parish,) Vermont, containing about thirteen hundred inhabitants, it appears that from 1797

to 1816 the whole number of deaths was four hundred and ten, of which number forty-nine, or one-eighth of the whole were by consumption. In the country there is, I apprehend, less predisposition to this disease than in cities, though exciting causes are in the former more numerous and powerful.

Has this disease increased in the United States within the last half century?—From my own observation, and from statements furnished me by aged medical men, I think it has considerably increased in country towns. In some of the cities it appears to have increased no faster than the population, while in others the increase of the disease has been much the greatest. This is particularly true of the city of New York, where in 1830 the deaths by consumption were 974, and in 1835 amounted to 1437.

Formerly, as we are informed by Dr. Colden, there was but little of the disease in New York. Speaking of the city about ninety years since, he observes: "The air of the country being always clear, we have but few consumptions, or diseases of the lungs. Persons inclined to be consumptive in England, are often perfectly cured here by our fine air." Similar observations respecting the rarity of consumptive diseases in this country, and the beneficial effects of our climate upon those who came here from Europe with impaired health, are found in the letters and writings of the first settlers of New England. Probably some recoveries were attributed to the influence of the climate that should have been credited to other causes. That the disease, however, was less common half a century since than at the present time, is evident from the observations of aged medical men. Dr. Holyoke of Salem, in a letter to Professor Wigglesworth, in 1790, observes: "This disease has of late become much more frequent, and makes up now, I believe, a tenth or perhaps a sixth of our whole bill of mortality." Consumption has, however, always prevailed here. It was the most fatal disease among the Indians, previous to the settlement of this country by the Europeans, and since then is said to have become still more destructive. The celebrated Indian chief, Red Jacket, has lost nine of his family by consumption.

*The Knickerbocker New York Monthly Magazine, July* 1836.

